# A cross-sectional study of the relationship between sexual compulsivity and unprotected anal intercourse among men who have sex with men in shanghai, China

**DOI:** 10.1186/s12879-018-3360-x

**Published:** 2018-09-15

**Authors:** Xin Wang, Zezhou Wang, Xueqin Jiang, Rui Li, Ying Wang, Gang Xu, Huachun Zou, Yong Cai

**Affiliations:** 1 School of Public Health, Shanghai Jiao Tong University, School of Medicine, Shanghai, People’s Republic of China; 2 Department of Public Health Science, School of Medicine and Dentistry, University of Rochester, New York, USA; 3 School of Public Health (Shenzhen), Sun Yat-sen University, Shenzhen, People’s Republic of China; 4 School of Public Health, Sun Yat-sen University, Guangzhou, People’s Republic of China; 5 Kirby Institute, University of New South Wales, Sydney, Australia

**Keywords:** Men who have sex with men, Sexual compulsivity, Unprotected anal intercourse, Sex partners

## Abstract

**Background:**

HIV prevalence among men who have sex with men (MSM) in China is rising rapidly, and unprotected anal intercourse (UAI) is associated with HIV transmission. Recent research has shown that associations between UAI and other factors can differ according to the type of sex partners, including regular partners and casual partners. This study aimed to explore the relationship between sexual compulsivity and UAI according to partner type among MSM in Shanghai, China.

**Methods:**

A cross-sectional study was conducted among 547 MSM from four districts in Shanghai, China. All participants were recruited using snowball sampling. The Sexual Compulsivity Scale was used to evaluate participants’ sexual compulsivity. Multivariable logistic regression was used to identify factors associated with sexual compulsivity and UAI. The mediation effects of substance use before sex on the relationship between sexual compulsivity and UAI were tested through mediation analyses.

**Results:**

After adjusting for sociodemographic variables, sexual compulsivity was associated with overall UAI (adjusted odds ratios [AOR] = 1.039, 95% confidence intervals [CI] = 1.004–1.075), UAI with non-regular sex partners (AOR = 1.089, 95% CI = 1.033–1.148) and UAI with commercial sex partners (AOR = 1.185, 95% CI = 1.042–1.349). No significant association was found between sexual compulsivity and UAI with regular sex partners (AOR = 1.029, 95% CI = 0.984–1.077). Mediation analyses indicated that the relationship between sexual compulsivity and UAI was not mediated by either alcohol use before sex or drug use before sex.

**Conclusions:**

The association between sexual compulsivity and UAI varies depending on the type of UAI partner. Therefore, individuals may engage in different types of UAI for different reasons, and tailored HIV cognitive–behavioral intervention programs are needed.

## Background

HIV transmission in China occurs in various ways, including intravenous drug use, blood or plasma transfusion, and high-risk sexual behaviors, particularly among men who have sex with men (MSM) [1]. Among people living with HIV (PLWH) in China, the approximate percentage of infections from unprotected male-to-male sexual contact was 7.3, 11.0, 14.7, and 17.4% in 2005, 2007, 2009, and 2011, respectively [2–5]. Related data suggest that the fastest increase in HIV transmission in China is found in MSM [6, 7]. MSM have a disproportionately high HIV prevalence, which can be ascribed to the high prevalence of unprotected anal intercourse (UAI) [8, 9], one of the riskiest sexual behaviors for HIV transmission [10–13] in this subpopulation. Therefore, an in-depth understanding of UAI is urgently needed to prevent the rapid spread of HIV among MSM. There are many factors related to UAI, such as drug use [14, 15], depressive symptoms [16], lower risk of perception of UAI [16, 17], non-disclosure of sexual orientation to parents [18], self-efficacy in condom use [19], sexual sensation seeking [15, 20], and sexual compulsivity [19, 21–25].

Sexual compulsivity is “an insistent, repetitive, intrusive, and unwanted urge to perform specific acts often in ritualized or routinized fashions” [24], which is characterized by sexual fantasies and can interfere with personal, interpersonal, and vocational activities [26–28]. Individuals who are incapable of controlling sexual impulses sufficiently and are preoccupied with sexual activities may tend to engage in high-risk sexual behaviors disregarding the probability of contracting HIV and other potential adverse consequences [29–31]. To assess the degree of sexual compulsivity, Kalichman and colleagues developed the 10-item Sexual Compulsivity Scale (SCS), which was based on a self-assisted guide for self-reported sexual addiction [24, 32–34]. This scale has been widely used and shown to be reliable among sexually active individuals, including MSM and heterosexual men and women [15, 24, 34–37]. High sexual compulsivity, in many studies, has been certified that corresponded to high-risk sexual behaviors in MSM [38, 39]. For MSM with different ethnic and racial backgrounds, sexual compulsivity has been recognized as a stable personality trait [40]. The SCS has been translated into Chinese and back-translated into English by Chinese researchers to verify its reliability and validity [36]. The validated Chinese version of sexual compulsivity scale used in the present study can also be applied in many other populations in China as long as they can read and write the same Chinese language [20].

Many previous studies have found a significant association between UAI and sexual compulsivity [19, 21–25]. Some research has examined this high-risk sexual behavior in relation to the type of sexual partner with whom participants practice UAI [20, 36, 41–46]. These studies have found variation in the relationships between independent variables and different types of UAI (including UAI with regular sex partners, UAI with casual sex partners, and UAI with commercial sex partners). In the meantime, some survey studies found the prevalence rates of different types of UAI vary [42, 45–49]. Wang et al. (2017) suggested that cognitive variables, psychological factors, emotion-related variables, and social-structural factors are strongly associated with UAI with regular and/or non-regular sexual partners [41]. Therefore, research on the relationship between sexual compulsivity and UAI according to partner type may help to inform partner type-specific HIV prevention strategies that target MSM. In addition, substance use has been recognized as a robust predictor of UAI [14, 15] and a mediator of the association between sexual compulsivity and UAI [21]. Therefore, testing for mediation by substance use before sex was conducted to understand whether the relationship between sexual compulsivity and UAI is mediated by substance use.

We conducted this cross-sectional study in Shanghai, China, and evaluated relationships between sexual compulsivity and different types of UAI. The main hypotheses were 1) sexual compulsivity is associated with UAI, and 2) the relationship between sexual compulsivity and UAI varies according to partner type.

## Methods

### Setting, sample and recruitment

Shanghai, a large cosmopolitan city with relatively more tolerance to people with diversified sexuality, MSM in particular, making it an appropriate social setting for studies targeting MSM. This cross-sectional study used a snowball sampling method to recruit eligible participants from the Changning, Jingan, Zhabei, and Pudong districts from March 2014 to August 2014. This method initially identifies subgroup members from whom the targeted data can be collected; then these initial members serve as “seed” to recruit new eligible participants. These participants, in turn, are encouraged to recruit other new participants until the sample size reaches the goal. Eligibility criteria in this research included male gender, age above 16 years, and having had UAI with another man in the past 6 months. With the help of the local Center for Disease Control and Prevention and some non-government organizations, 5 to 10 eligible persons from each district were enrolled as “seeds”. A total of 547 eligible participants were enrolled. Each participant signed an informed consent form before completing a questionnaire. Participants received 100 CNY (about 15.5 USD) as compensation. Trained workers introduced the survey to participants and answered any questions they had. Subsequently, anonymous face-to-face interviews were carried out to help participants to complete a series of questionnaires collecting sociodemographic data, data on behavioral variables, and SCS scores. At the end of this process, one participant’s data were excluded because he had not specified the partner type in his response.

### Ethics, consent, and permissions

Each participant provided written, informed consent before participation. This study strictly complied with American Psychological Association standards and was approved by the institutional review board of the Shanghai Jiao Tong University School of Public Health.

### Measures

Questionnaire data on sociodemographics, behavioral variables, and total SCS scores comprised the independent variables.

#### Sociodemographics

Respondents were asked about their age, highest educational level, current marital status (with women), monthly salary, residential status, and self-reported sexual orientation.

#### Behavioral variables

Behavioral variables measured were overall UAI and different forms of UAI according to partner type in the past 6 months, as well as substance use before sex. Individuals who reported inconsistent condom use (any at all, over the last 6 months) during sex with men were coded as having had UAI with male sex partners; this operational recording has been commonly used in published studies [50, 51]. Information about the type of sexual partner was also obtained. Regular sex partners were defined as boyfriends; namely, those individuals in stable relationships with participants. Non-regular sex partners were defined as sexual partners who were neither regular nor commercial. Commercial sex partners were defined as partners receiving money from participants for transactional sex. Some published studies on sexual activities have used similar definitions for sex partner types [52–54].

#### Sexual compulsivity

The degree of sexual compulsivity was assessed using the SCS, a 10-item, four-point Likert-type scale ranging from 1 (strongly disagree) to 4 (strongly agree). The total score ranges from 10 to 40. Sample items included “My sexual appetite has gotten in the way of my relationships,” “My sexual thoughts and behaviors are causing problems in my life” and “I sometimes fail to meet my commitments and responsibilities because of my sexual behaviors.” A higher total score indicates a greater degree of sexual compulsivity. Cronbach’s α for this scale is 0.86, as reported by Kalichman & Rompa [24], and was 0.853 for the current sample.

### Statistical analysis

Internal reliability was assessed by using the Cronbach’s α. Descriptive analysis was performed, then the associations between background variables and sexual compulsivity were examined using t-tests and ANOVA. In addition, multivariable logistic regression was conducted to determine the association between independent variables and different types of UAI, obtained their adjusted odds ratios (AOR) and 95% confidence intervals (CI). The criterion of statistical significance was *p* < .05. At the final stage, meditational analyses were conducted by computing the separate *Z*_Mediation_, which was recommended by a published study for categorical mediators and dependent variables [55]. All data analyses were performed using SPSS version 22.0 for Windows (SPSS, Inc., Chicago, IL, USA).

### Mediation analyses

The aims of this research included investigating whether substance use before sex as a robust predictor of UAI mediate the relationship between sexual compulsivity and UAI. According to a published study recommending the solution for meditational analyses using categorical mediators and dependent variables, the *Z*_*Mediation*_ was computed [55]. The mediation effect is significant at the level of α = 0.05 if the *Z*_*Mediation*_ exceeds |1.96| (for a 2-tailed test with α = 0.05).

## Results

### Sample description

Table 1 shows the frequency distribution of participant sociodemographic characteristics and Table 2 shows descriptive statistics for sexual compulsivity. Most respondents were single non-local people aged 25–40 years, with a college-level education or above and self-reported as gay/homosexual. The distribution of income was even. Regarding the substance use, 49.3% of participants reported alcohol use before sex, and 96.9% of participants reported no drug use before sex during the 6 months prior to the study. Of the participants, 54.4% were coded as having had UAI with male sex partners in the past 6 months. Regarding sex partners, 61.5% of respondents reported having regular sex partners and 50.9% of these had had UAI with regular sex partners in the past 6 months; 51.8% of respondents reported having non-regular sex partners and 42.8% of these had had UAI with non-regular sex partners in the past 6 months; 14.3% of respondents reported having commercial sex partners and 55.1% of these had had UAI with commercial sex partners in the past 6 months. The range, mean and median of participants’ SCS scores were 30, 22.41 and 23.00 respectively.

**Table 1:** Frequency of sociodemographic characteristics, sexual partner type, and unprotected anal intercourse types (*N* = 546)

Variables	*N* (%)
Age group (years)	
< 25	148 (27.1)
25–40	336 (61.5)
> 40	62 (11.4)
Highest educational level	
Senior high school or below	157 (28.8)
College degree or above	389 (71.2)
Current marital status	
Married	82 (15.0)
Single	433 (79.3)
Divorced or widowed	31 (5.7)
Income (monthly CNY)	
< 3000	133 (24.4)
3000–6000	211 (38.6)
> 6000	202 (37.0)
Residencial status	
Local	147 (26.9)
Non-local	399 (73.1)
Self-reported sexual orientation	
Non-homosexual	157 (28.8)
Gay/homosexual	389 (71.2)
Alcohol use before sex	
No	277 (50.7)
Yes	269 (49.3)
Drug use before sex	
No	529 (96.9)
Yes	17 (3.1)
Have regular sex partners	
Yes	336 (61.5)
No	210 (38.5)
UAI with regular sex partners	
Yes	171 (50.9)
No	165 (49.1)
Have non-regular sex partners	
Yes	283 (51.8)
No	263 (48.2)
UAI with non-regular sex partners	
Yes	121 (42.8)
No	162 (57.2)
Have commercial sex partners	
Yes	78 (14.3)
No	468 (85.7)
UAI with commercial sex partners	
Yes	43 (55.1)
No	35 (44.9)

**Table 2:** Descriptive statistics for sexual compulsivity (*N* = 546)

Sociodemographics	Sexual Compulsivity Scale score	
	Mean ± SD	p^a^
Age group (years)		
< 25	21.62 ± 5.34	0.082
25–40	22.78 ± 5.18	
> 40	22.34 ± 5.24	
Highest educational level		
Senior high school or below	23.55 ± 5.07	0.001
College degree or above	21.96 ± 5.25	
Current marital status		
Single	22.09 ± 5.27	0.001
Married	24.44 ± 4.72	
Divorced or widowed	21.61 ± 5.10	
Income (monthly CNY)		
< 3000	22.74 ± 5.11	0.596
3000–6000	22.45 ± 5.43	
> 6000	22.15 ± 5.15	
Residencial status		
Local	21.38 ± 5.14	0.005
Non-local	22.79 ± 5.24	
Self-reported sexual orientation		
Non-homosexual	22.36 ± 4.76	0.871
Gay/homosexual	22.44 ± 5.44	
Alcohol use before sex		
No	22.08 ± 5.16	0.126
Yes	22.76 ± 5.32	
Drug use before sex		
No	22.29 ± 5.18	0.003
Yes	26.12 ± 5.95	
UAI with regular sex partners		
Yes	21.82 ± 4.96	0.441
No	21.39 ± 5.12	
UAI with non-regular sex partners		
Yes	23.92 ± 4.56	0.003
No	22.05 ± 5.50	
UAI with commercial sex partners		
Yes	25.19 ± 4.58	0.027
No	22.83 ± 4.62	

*UAI* unprotected anal intercourse

^a^t-test or ANOVA

Table 2 shows total SCS scores by sociodemographic and behavioral variables. There were significant between-group differences in SCS scores for highest educational level, current marital status, residential status, UAI with non-regular sex partners, and UAI with commercial sex partners. Individuals having had UAI with non-regular sex partners and with commercial sex partners have a higher SCS mean scores than individuals having had UAI with regular sex partners.

### Relationships between background variables and UAI, UAI with regular sex partners, UAI with non-regular sex partners, and UAI with commercial sex partners

Analyses showed that highest educational level and monthly salary were significantly related to UAI. Age was significantly related to UAI with regular sex partners. Age, highest educational level, and self-reported sexual orientation were significantly related to UAI with non-regular sex partners. Self-reported sexual orientation was significantly related to UAI with commercial sex partners. Table 3 presents the main outcome of the analysis.

**Table 3:** Relationships between sociodemographics, sexual compulsivity, and UAI/UAIRP/ UAINP/UAICP

Sociodemographics	UAI (*N* = 546)		UAIRP (*N* = 336)		UAINP (*N* = 283)		UAICP (*N* = 78)	
	N (%)	AOR (95% CI)	N (%)	AOR (95% CI)	N (%)	AOR (95% CI)	N (%)	AOR (95% CI)
Age group (years)								
< 25	76 (51.4%)	1	53 (58.9%)	1	19 (29.2%)	1	10 (62.5%)	1
25–40	186 (55.4%)	1.011 (0.654–1.563)	96 (47.3%)	0.517 (0.295–0.906)*	84 (45.4%)	2.176 (1.097–4.316)*	24 (53.3%)	0.517 (0.125–2.139)
> 40	35 (56.5%)	1.076 (0.530–2.181)	22 (51.2%)	0.595 (0.245–1.444)	18 (54.5%)	3.011 (1.049–8.639)*	9 (52.9%)	0.621 (0.099–3.877)
Highest educational level								
Senior high school or below	99 (63.1%)	1	47 (50.0%)	1	53 (59.6%)	1	27 (57.4%)	1
College degree or above	198 (50.9%)	0.614 (0.387–0.974)*	124 (51.2%)	0.957 (0.529–1.732)	68 (35.1%)	0.418 (0.218–0.799)**	16 (51.6%)	1.335 (0.367–4.862)
Current marital status								
Single	232 (53.6%)	1	139 (51.1%)	1	83 (39.5%)	1	25 (56.8%)	1
Married	48 (58.5%)	0.803 (0.458–1.409)	22 (45.8%)	0.841 (0.410–1.725)	26 (47.3%)	0.460 (0.211–1.002)	13 (52.0%)	0.328 (0.079–1.356)
Divorced or widowed	17 (54.8%)	0.795 (0.354–1.788)	10 (62.5%)	1.712 (0.561–5.224)	12 (66.7%)	1.700 (0.538–5.368)	5 (55.6%)	0.782 (0.132–4.643)
Income (monthly CNY)								
< 3000	64 (48.1%)	1	37 (45.7%)	1	28 (48.3%)	1	19 (61.3%)	1
3000–6000	132 (62.6%)	1.969 (1.235–3.139)**	70 (54.7%)	1.691 (0.933–3.064)	57 (47.5%)	1.151 (0.568–2.331)	16 (55.2%)	0.736 (0.213–2.538)
> 6000	101 (50.0%)	1.341 (0.803–2.240)	64 (50.4%)	1.567 (0.807–3.043)	36 (34.3%)	0.769 (0.347–1.708)	8 (44.4%)	0.260 (0.054–1.257)
Residential status								
Local	80 (54.4%)	1	52 (52.5%)	1	27 (42.2%)	1	7 (43.8%)	1
Non-local	217 (54.4%)	0.854 (0.565–1.291)	119 (50.2%)	0.791 (0.472–1.326)	94 (42.9%)	0.982 (0.516–1.866)	36 (58.1%)	2.173 (0.556–8.503)
Self-reported sexual orientation								
Non-homosexual	91 (58.0%)	1	46 (50.0%)	1	46 (50.5%)	1	26 (65.0%)	1
Gay/homosexual	206 (53.0%)	0.837 (0.564–1.242)	125 (51.2%)	0.967 (0.580–1.615)	75 (39.1%)	0.571 (0.327–0.996)*	17 (44.7%)	0.222 (0.069–0.709)*
Sexual Compulsivity Scale score		1.039 (1.004–1.075)*		1.029 (0.984–1.077)		1.089 (1.033–1.148)**		1.185 (1.042–1.349)*

*AOR* adjusted odds ratio, *UAI* unprotected anal intercourse, *UAIRP* unprotected anal intercourse with regular sex partners, *UAINP* unprotected anal intercourse with non-regular sex partners, *UAICP* unprotected anal intercourse with commercial sex partners, *95% CI* 95% confidence interval

*p < 0.05, **p < 0.01

### Relationships between sexual compulsivity and UAI, UAI with regular sex partners, UAI with non-regular sex partners, and UAI with commercial sex partners

The relationships between sexual compulsivity and UAI, UAI with non-regular sex partners, and UAI with commercial sex partners were significant. AORs for the associations between sexual compulsivity and different types of UAI were calculated after adjusting for background variables. Sexual compulsivity was found to be associated with overall UAI (AOR = 1.039, 95% CI = 1.004–1.075), UAI with non-regular sex partners (AOR = 1.089, 95% CI = 1.033–1.148) and UAI with commercial sex partners (AOR = 1.185, 95% CI = 1.042–1.349). No significant association was found between sexual compulsivity and UAI with regular sex partners (AOR = 1.029, 95% CI = 0.984–1.077).

After adjusting for the effects of background variables, the results showed that for each unit increase in the total SCS score, the odds of having had UAI increased by 3.9%, the odds of having had UAI with non-regular sex partners increased by 8.9% and the odds of having had UAI with commercial sex partners increased by 18.5%. Given the range of the total SCS score, these increases in odds are considerable. Table 3 presents the main outcome of this analysis.

### Mediation analyses

The meditational analyses indicated that the relationships between sexual compulsivity and UAI, UAINP, UAICP were not mediated by either alcohol use before sex or drug use before sex. Table 4 presents the main outcomes of the analyses.

**Table 4:** Summary of analyses testing mediation

Independent variable	Dependent variable	Mediator	*Z*_*Mediation*_	*Significance*
Sexual compulsivity	UAI	Alcohol use before sex	−1.055452192	ns
Sexual compulsivity	UAI	Drug use before sex	−1.3142261	ns
Sexual compulsivity	UAINP	Alcohol use before sex	−1.034265068	ns
Sexual compulsivity	UAINP	Drug use before sex	1.026276748	ns
Sexual compulsivity	UAICP	Alcohol use before sex	0.62461196	ns
Sexual compulsivity	UAICP	Drug use before sex	0.436276904	ns

*UAI* unprotected anal intercourse, *UAINP* unprotected anal intercourse with non-regular sex partners, *UAICP* unprotected anal intercourse with commercial sex partners

*Z*_*Mediation*_ < −1.96 or *Z*_*Mediation*_ > 1.96

## Discussion

This survey explored the relationships between sexual compulsivity and different types of UAI among MSM in Shanghai, China. The prevalence rates for different types of UAI among participants were 50.9% (UAI with regular sex partners), 42.8% (UAI with non-regular sex partners), and 55.1% (UAI with commercial sex partners). These statistics are in line with previous study [42, 45–49], indicating that the prevalence rate of UAI with regular sex partners is higher than the prevalence rate of UAI with non-regular sex partners. The findings also showed that the association between sexual compulsivity and UAI varied according to partner type. In other words, sexual compulsivity was significantly associated with UAI in general, UAI with non-regular sex partners, and UAI with commercial sex partners. No significant association was observed between sexual compulsivity and UAI with regular sex partners. This result is consistent with findings from several previous studies, suggesting that individuals who exhibit a greater degree of sexual compulsivity are more likely to engage in UAI with casual sex partners than those who exhibit less sexual compulsivity [17, 23, 32–34, 56]. In addition, we investigated potential mediators of the relationships between sexual compulsivity and UAI, UAINP, UAICP, and failed to find any significant mediation effect. More research is warranted to understand whether substance use before sex mediates the association between sexual compulsivity and UAI in Chinese MSM.

The choice of variable type (categorical variable versus continuous variable) is a critical issue that could potentially influence the result of statistical analyses. Before presenting results produced by using the continuous SC variable in Table 3, multivariable analyses were carried out respectively to compare the results obtained by using the continuous SCS variable and by using the categorical SCS variable. In despite of a lack of established, defined cut-point to designate sexual compulsivity, the developers of this scale used the 80th percentile as their cut-point to ensure that compulsive individuals defined by them were at least one SD (standard deviation) above the mean on this scale [21]. The 85% percentile was defined as the cut-point in our study according to this method. The result obtained by using the categorical variable still failed to find a significant association between SC and UAI with regular sex partners while still finding evidence of association for the other partner types (general UAI and UAI with commercial sex partners). Given that the result may vary according to different cut-points and the cut-point may vary according to different samples, using the continuous variable may produce a more stable result.

Analyses indicated that highest educational level and monthly salary were significantly related to UAI; age was significantly related to UAI with regular sex partners; age, highest educational level, and self-reported sexual orientation were significantly related to UAI with non-regular sex partners; self-reported sexual orientation was significantly related to UAI with commercial sex partners. Participants with a higher educational level were less likely to perform UAI. This difference may result from the situation that participants with a lower educational level are less informed about HIV prevention knowledge in China [57]. Therefore, sex and HIV/AIDS-related education and research are urgently needed, not only to fill the knowledge gap in Chinese sex education but also to help mitigate social discrimination and stigma toward MSM [58].

The differences in the associations between sexual compulsivity and UAI with regular sex partners, UAI with non-regular sex partners, and UAI with commercial sex partners provide new insights into the reasons for different UAI and indicate the importance of differentiating between these practices in future research [41]. Continued research on the nature of sexual compulsivity may help to clarify the mechanism underlying UAI with non-regular and commercial sex partners. Sexual compulsivity represents sexual preoccupation and lack of sexual control, which is more likely to be associated with casual sexual interactions [32, 34]. This may be a result of a diminished ability to avoid sexual risk, as rational decision-making may be impaired under sexual arousal, making sexual risks less salient [59]. In other words, individuals who are sexually aroused may have a compromised capacity in perceiving specific risky sexual behaviors and avoid them. Therefore, individuals with a high level of sexual compulsivity may show a diminished long-term ability to avoid risky sexual behaviors, as such individuals experience prolonged states of sexual arousal [59]. However, although there is a relatively high prevalence rate of UAI with regular sex partners, it seems not to be a result of an impaired ability to avoid sexual risks. Crawford et al. (2006) reported that with regular partners who are HIV-seropositive, insertive UAI without ejaculation is much more frequent than receptive UAI with ejaculation, whereas with casual partners who are HIV-seropositive, insertive and receptive UAI practices occur almost as frequently [46]. Therefore, it is possible that individuals who practice UAI with regular sex partners are not unaware of the HIV risk. Previous studies on regular sex partners have suggested several important factors related to UAI with regular sex partners, including greater sexual impulsivity and concern about perceptions of mistrust between partners, intimacy interference, and syndemic stress [47, 60–62].

Thus, factors related to UAI should be considered in light of participants’ partner types, and HIV prevention strategies should be tailored to specific types of UAI, which is in line with previous research recommendations [41, 63]. For UAI with non-regular and commercial sex partners, therapy for sexual compulsivity may be effective to promote sexual health. Furthermore, providing condoms, communication, and behavior change can help to decrease UAI exposure [45]. Regarding UAI with regular sex partners, pre-exposure prophylaxis is a promising way to prevent HIV transmission among MSM individuals who are willing to practice condomless sex with partners to maintain intimacy [64]. However, a baseline survey for a clinical trial of PrEP in Shanghai indicated that the actual willingness of MSM to participate in the PrEP program is low [65]. At current circumstance in China, the implementation of PrEP is still challenging, and effective education to promote acceptance of PrEP is needed.

Several limitations of this study should be pointed out. First, caution is needed in drawing a causal conclusion, as this was a cross-sectional study. Second, the snowball sampling method may have caused selection bias, which might have affected the accuracy of the study conclusions; however, this sampling method is frequently used in studies targeting hard-to-reach populations. Additionally, social desirability may have affected the responses, as the questionnaire surveys were completed with the help of face-to-face interviews; participants thus may have been reluctant to provide honest answers. Finally, the HIV serostatus of participants and the type of sexual behavior (e.g., insertive or receptive) were not measured in this study.

## Conclusions

Our study showed that the association between sexual compulsivity and UAI varies according to the type of UAI. Sexual compulsivity is not significantly associated with UAI with regular sex partners but is significantly associated with UAI with non-regular and commercial sex partners. Tailored cognitive–behavioral therapies targeting various types of UAI are urgently needed to optimize current HIV intervention programs.

## Abbreviations

AOR: Adjusted odds ratio
CI: Confidence interval
HIV: Human immunodeficiency virus
MSM: Men who have sex with men
ORu: Univariate odds ratio
SCS: Sexual Compulsivity Scale
UAI: Unprotected anal intercourse
